# Gene Expression Profiling of Solitary Fibrous Tumors

**DOI:** 10.1371/journal.pone.0064497

**Published:** 2013-05-29

**Authors:** François Bertucci, Corinne Bouvier-Labit, Pascal Finetti, Philippe Metellus, José Adelaide, Karima Mokhtari, Dominique Figarella-Branger, Anne-Valérie Decouvelaere, Catherine Miquel, Jean-Michel Coindre, Daniel Birnbaum

**Affiliations:** 1 Département d'Oncologie Moléculaire, Centre de Recherche en Cancérologie de Marseille (CRCM), Institut Paoli-Calmettes (IPC), UMR1068 Inserm; Marseille, France; 2 Département d'Oncologie Médicale, IPC, CRCM, UMR1068 Inserm, Marseille, France; 3 Faculté de Médecine, Aix-Marseille Université, Marseille, France; 4 Département d’Anatomopathologie, Hôpital de la Timone, Marseille, France; 5 Département de Neurochirurgie, Hôpital de la Timone, Marseille, France; 6 Département de Neuropathologie, Hôpital Pitié Salpétrière, Paris, France; 7 Département d’Anatomopathologie, Centre Léon Bérard, Lyon, France; 8 Département de Neuropathologie, Hôpital Sainte Anne, Paris, France; 9 Département d’Anatomopathologie, Institut Bergonie, Bordeaux, France; University of Alabama at Birmingham, United States of America

## Abstract

**Background:**

Solitary fibrous tumors (SFTs) are rare spindle-cell tumors. Their cell-of-origin and molecular basis are poorly known. They raise several clinical problems. Differential diagnosis may be difficult, prognosis is poorly apprehended by histoclinical features, and no effective therapy exists for advanced stages.

**Methods:**

We profiled 16 SFT samples using whole-genome DNA microarrays and analyzed their expression profiles with publicly available profiles of 36 additional SFTs and 212 soft tissue sarcomas (STSs). Immunohistochemistry was applied to validate the expression of some discriminating genes.

**Results:**

SFTs displayed whole-genome expression profiles more homogeneous and different from STSs, but closer to genetically-simple than genetically-complex STSs. The SFTs/STSs comparison identified a high percentage (∼30%) of genes as differentially expressed, most of them without any DNA copy number alteration. One of the genes most overexpressed in SFTs encoded the ALDH1 stem cell marker. Several upregulated genes and associated ontologies were also related to progenitor/stem cells. SFTs also overexpressed genes encoding therapeutic targets such as kinases (*EGFR*, *ERBB2*, *FGFR1*, *JAK2*), histone deacetylases, or retinoic acid receptors. Their overexpression was found in all SFTs, regardless the anatomical location. Finally, we identified a 31-gene signature associated with the mitotic count, containing many genes related to cell cycle/mitosis, including *AURKA*.

**Conclusion:**

We established a robust repertoire of genes differentially expressed in SFTs. Certain overexpressed genes could provide new diagnostic (*ALDH1A1*), prognostic (*AURKA*) and/or therapeutic targets.

## Introduction

Solitary fibrous tumor (SFT) is a rare spindle cell neoplasm, observed at all ages, but more often between 50 and 70 years [1]. Initially, SFTs were most commonly found in the pleura [1], but may in fact occur in any part of the body [2]. The cell-of-origin and etiology are uncertain, but SFTs are thought to derive from mesenchymal fibroblastic cells. According to the WHO classification of Soft Tissue Tumors, SFTs have a “patternless architecture characterized by a combination of alternating hypocellular and hypercellular areas separated from each other by thick bands of hyalinized somewhat keloidal, collagen and branching haemangiopericytoma-like vessels”. These tumors have variable immunoreactivity for non specific markers such as CD34, CD99, BCL2, and vimentin. Misdiagnosis is frequent with other spindle cell lesions, especially synovialosarcomas or fibrosarcomas [3], notably in the case of unusual location. Prognosis is good in the majority of cases. However, 10 to 20% of SFTs (so-called “malignant SFTs”) behave aggressively with local invasiveness and/or recurrences, and/or occasional distant metastases [4]; [5]. Histological features for malignancy include large size, high mitotic count (>4 per 10 high-power fields, HPF), high cellularity, necrosis, hemorrhage, cytological atypias with pleiomorphism, and infiltrative growth pattern [4]; [6]. But, these features do not always predict unfavorable clinical outcome [2]; [4]; [7]; [8]. Today, the clinical outcome of SFTs is difficult to predict, and efforts are ongoing to improve the prognostication of disease [9]. For many authors, the complete resection of the tumor with negative margins is the most important prognostic factor. Surgery represents the mainstay of treatment for primary tumors or local relapses. In the case of an unresectable or metastatic disease, chemotherapy and radiotherapy are not efficient and the prognosis is poor [10]. Clearly, more reliable diagnostic and prognostic markers, and alternative treatments are urgently needed for SFT patients.

At the molecular level, the disease is poorly known. No consistent cytogenetic abnormality has been reported [11]–[16]. Frequent expression of PDGFR and MET tyrosine kinase receptors was reported [17], as well as rare mutations involving *PDGFRB*. P53 expression has been associated with poor prognosis [17]; [18] as well as *PDGFRB* mutation [19]. Alterations of the IGF and insulin receptor pathway have been documented [20]. However, to date, none of these alterations has reached clinical application as diagnostic, prognostic or therapeutic target. High-throughput molecular analyses have been applied to soft tissue sarcomas (STSs) [21], but very rarely to SFTs, To our knowledge, only two gene expression profiling studies have been reported to date [22]; [23], including a relatively limited number of cases (13 and 23), and only one compared SFT with STS [22].

Here, we have hybridized a series of 16 SFTs. using whole-genome DNA microarrays and analyzed their gene expression profiles in combination with profiles of publicly available data sets including 36 additional SFTs and 212 STSs. We compared SFTs and STSs in terms of transcriptional heterogeneity and profiles. We also compared SFTs according to their anatomical location and mitotic index. Finally, immunohistochemistry (IHC) was applied to validate at the protein level the differential expression of some discriminating genes.

## Materials and Methods

### Gene Expression Profiling of Solitary Fibrous Tumors

Sixteen pre-treatment samples of pathologically confirmed SFT were available for RNA profiling. They were collected from 16 patients who underwent initial surgery (N = 12) and/or diagnostic biopsy (N = 4) in one of the 6 participating centers. Samples were macrodissected by pathologists, and frozen within 30 min of removal in liquid nitrogen in our biobank (Biobank authorization number 2008/70, APHM). All profiled specimens contained more than 70% of tumor cells. The main histoclinical characteristics of patients and samples are listed in Table 1. The median age was 52 years, and the sex ratio 7F/9M. Samples corresponded to primary tumors (14 cases) and local relapses (2 cases). Their origin was meningeal (12 cases) and extra-meningeal (soft tissue: 4 cases). The mitotic count was low (5 or less than 5 mitoses for 10 high-power fields HPF) in 10 samples, and high (more than 5 mitoses/10 HPF) in 6. Ten cases were cellular forms of SFT while six were conventional. Each patient gave written informed consent for molecular analysis, and the study was approved by our institutional ethics committee.

**Table 1:** Histoclinical characteristics of SFT samples.

Characteristics	N	%
Age, years		
median	52	
range	22–68	
Sex		
female	7	44
male	9	56
Location		
meningeal	12	75
extra-meningeal	4	25
Histological subtype		
cellular	10	62
fibrous	6	38
Mitotic count		
low	10	62
high	6	38

Total RNA was extracted from frozen samples by using the All-In-One Norgen Biotek kit (Thorold, Canada) and integrity was controlled by Agilent analysis (Bioanalyzer, Palo Alto, CA). Gene expression profiling was done with Affymetrix U133 Plus 2.0 human oligonucleotide microarrays containing over 47,000 transcripts and variants, including 38,500 well-characterized human genes. Preparation of cRNA, hybridizations, washes and detection were done as previously described [24]. Expression data were analyzed by the RMA (Robust Multichip Average) method in R using Bioconductor and associated packages [25]. RMA performed the background adjustment, the quantile normalization and finally the summarization of 11 oligonucleotides per gene. Raw data of the 16 SFT samples that we have hybridised are publicly available in a MIAME format in the ArrayExpress database (accession number: E-MTAB-1361).

### Public Gene Expression Data Sets

To increase the size of the SFT series and include STS samples as controls, we collected three publicly available data sets: West’s set [23] collected from (http://microarray-pubs.stanford.edu/tma-portal/DTF_SFTbreast) and including 13 SFTs (surgical specimen; disease stage not available) and 30 STS profiled using 42,000-element cDNA microarrays, Hajdu’s set [22] collected from (http///cbio.mskcc.org/Public/SFT) and including 23 SFTs (surgical specimen representing 9 primary tumors, 4 local relapses and 10 metastatic relapses) and 33 STSs profiled using Affymetrix U133A microarrays, and Barretina’s set [26] collected from NCBI GEO database (GSE21124) and including 149 STSs profiled using U133A microarrays. Both expression and histoclinical (Table S1) data were collected.

### Analysis of Gene Expression Data

Whole-genome mRNA expression profiles of 52 SFTs and 212 STSs were available for analysis. For the unsupervised analysis and the supervised analyses (except that centered on the mitotic index), samples were divided in a learning set including our series pooled with West and Barretina’s series (29 SFTs and 179 STSs) and an independent validation set (Hajdu’s series: 23 SFTs and 33 STSs). Before analysis, expression data generated from different technological platforms and laboratories were processed to eliminate potential biases. The three combined series of the learning set (West and Barretina’s series and our series) were pre-processed together. By contrast, the validation set (Hajdu’s series), which was analyzed separately, did not need further pre-processing of uploaded data.

Pre-processing of expression data included as first step the selection of genes unique and common to the three data sets. We mapped hybridization probes across the different technological platforms (Affymetrix U133A and U133 Plus 2.0, and Stanford cDNA microarrays). Affymetrix gene chips annotations were updated using NetAffx Annotation files (www.affymetrix.com; release from 01/12/2008). Stanford gene annotations were retrieved and updated using EntrezGene (*Homo sapiens* gene information db, release from 09/12/2008, ftp://ftp.ncbi.nlm.nih.gov/gene/). All probes were then mapped based on their EntrezGeneID. When multiple probes were mapped to the same GeneID, the one with the highest variance in a particular data set was selected to represent the GeneID. For redundant Affymetrix probe sets, those with an extension « _at », next « s_at », and followed by all other extensions were preferentially kept. This step retained a total of 10,138 unique genes common to the three series. The second step was the normalization of the three combined data sets for these 10,138 genes using Distance Weighted Discrimination (DWD) [27]. Analysis of this pooled set was then unsupervised and supervised.

Unsupervised analysis was done using hierarchical clustering in the learning set. Before analysis, data were log_2_-transformed. Clustering was done using the Cluster program [28] with data median-centered on genes, Pearson correlation as similarity metrics and centroid linkage clustering. Results were displayed using TreeView program [28]. The robustness of tumor clusters was estimated by the AU (Approximately unbiased) p-values provided by multiscale bootstrap resampling in the R-package pvclust [29], larger the p-values, more robust the clusters.

Supervised analyses used a learning set and a validation set as recommended. The series used as learning or validation set in the different supervised analyses are indicated in Table S1. For each signature, Significance Analysis of Microarrays (SAM) [30] identified and ranked genes discriminating the two groups of samples in the learning set, from which PAM (Prediction Analysis of Microarrays) [31] developed a predictive model. The robustness of PAM models was estimated using both internal cross-validation (CV) across 100 iterations and external validation in an independent data set. For the mitotic index signature that included only overexpressed genes, we could not use PAM to develop the classifier. This latter was defined as a metagene computed as the mean expression level of included genes. The optimal threshold for classification was determined in the learning set using ROC curve (defined as the cut-off that maximizes the ROC area), and was then applied to the validation set (West’s series here).

To help in the interpretation, the lists of differential genes were interrogated using the Ingenuity Pathway Analysis (IPA) software (version 5.5.1–1002; Ingenuity Systems, Rewood City, CA) and the GO (Gene Ontology) database.

### Array-based Comparative Genomic Hybridization

We have previously reported an array-based comparative genomic hybridization (aCGH) analysis of 47 SFTs [32], including 12 out of the 16 SFT samples profiled in the present study at the transcriptional level. Samples had been profiled usng high-resolution 244K CGH microarrays (Hu-244A, Agilent Technologies, Massy, France), using a pool of 13 normal male DNA as reference. Here, we searched for copy number alterations (CNA) of the genes found as differentially expressed between SFTs and STSs. Low-level CNAs were defined as gain when log_2_ ratio was superior to 0.5 and as loss when inferior to −1.

### Immunohistochemistry

Automated immunohistochemistry (iHC) was performed on slides of tissue microarrays (TMA) paraffin blocks including 93 SFTs (80 meningeal and 13 from soft tissue) and 752 STSs including 98 genetically-confirmed synovial sarcomas, 50 epithelioid sarcomas, 44 dedifferentiated liposarcomas, 339 pleiomorphic or undifferentiated sarcomas, 71 leiomyosarcomas, and 150 GIST. TMA included also 147 « benign tumors » including 80 desmoId tumors and 31 pleiomorph/fusiform cell lipomas.

All tumor specimens were fixed in 4% formalin. TMAs were prepared as previously described [33]. For each sample, three representative sample areas were carefully selected from a hematoxylin–eosin-stained section of a donor block. The diameter of core cylinders was 0.6 mm for SFTs and 1 mm for soft tissue tumors. Each were punched from three representative areas and deposited into two separate recipient paraffin blocks using a specific arraying device (Alphelys). Automated IHC was performed using a Ventana automate (Benchmark XT, Ventana Medical Systems SA, Illkirch, France). Two proteins were analyzed: ALDH1 and AURKA, using respectively the anti-ALDH1 antibody (clone 44/ALDH, BD Transduction Laboratories, dilution: 1/500) and the anti-AURKA antibody (clone JLM2P, Novocastra, dilution 1/50). For ALDH1, the positive external control was a glioblastoma tissue sample, whereas negative controls corresponded to omission of primary antibody or irrelevant antibodies of the same isotype. IHC was recorded as positive when a cytoplasmic staining was observed in 5% or more of the tumor cells as previously described [34]. Then a semi-quantitative analysis was done for positive specimens leading to 3 categories: weak positivity (5–10% of stained cells), moderate positivity (11–50%), and strong positivity (>50%). For AURKA, the staining was recorded as positive when observed in at least 1% of tumor cells [35]; [36]. The positive control was lymphocytes in the germinal center of tonsil, whereas negative control was obtained by omitting the primary antibody.

### Statistical Analyses

Correlations between sample groups and histoclinical parameters were calculated with the Fisher’s exact test. Odds ratios (OR) were given with their 95% confidence interval. All statistical tests were two-sided at the 5% level of significance. Statistical analyses were done using the SPSS software (version 10.0.5).

## Results

### SFTs are Very Different from STSs on a Whole-genome Scale

**Figure 1:**
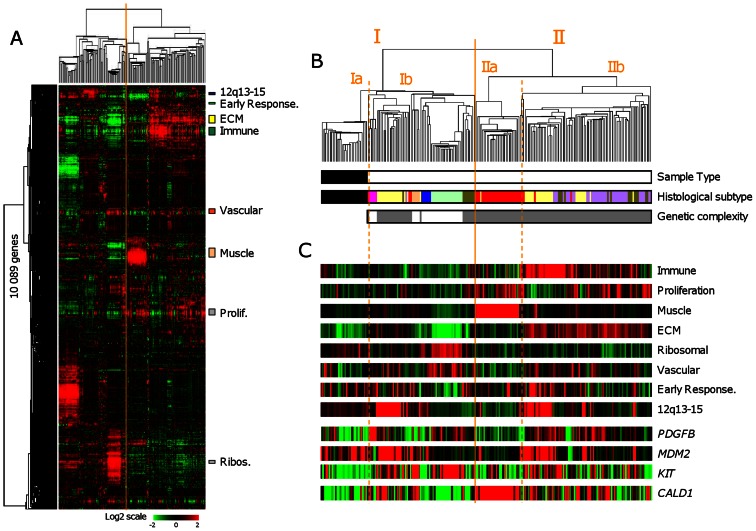
Whole-genome expression profiling of SFTs and STSs. *****A.***** Hierarchical clustering of 208 samples (29 SFTs and 179 STSs) and 10,089 genes with significant variation in mRNA expression level across the samples (SD≥0.25). Each row of the data matrix represents a gene and each column a sample. Expression levels are depicted according to the color scale shown at the bottom, where red and green indicate expression levels respectively above and below the median and the color saturation represents the magnitude of deviation from the median. The dendrogram of samples (above matrixes) represents overall similarities in gene expression profiles and is zoomed in B. Colored bars to the right indicate the locations of 8 gene clusters of interest. ***B.*** Dendrogram of samples. *Top*, two large groups of samples (designated I and II) are evidenced by clustering and delimited by the orange vertical line. Each cluster is divided into two subclusters (a and b) delimited by orange dotted vertical lines. *Bottom*, some characteristics of samples are represented according to a color ladder: sample type (black, SFT; white, STS); histological subtype (black, SFT; pink, DFSP; salmon, GIST; dark blue, synovial sarcoma; light green, myxoid/round cells liposarcoma; yellow, dedifferentiated liposarcoma; dark green, pleomorphic liposarcoma; red, leiomyosarcoma; purple, malignant fibrous histiocytoma); degree of genetic complexity of STSs (white, simple; grey, complex). ***C.*** Metagene (MG) of the 8 gene clusters shown in Figure 1A (the metagene is the mean expression level of included genes) and four control genes (EntrezGene symbol) known as differentially expressed according to the histological subtype.

Hierarchical clustering was applied to the 29 SFTs and 179 STSs pooled from the learning set (Figure 1A–B). All SFTs clustered together, suggesting clear distinction from STSs, and displayed relatively homogeneous expression profiles. In fact, all 208 samples were sorted into two major clusters (I and II), which correlated with the histological type (SFT, STS with simple genetic alterations thereafter designed genetically-simple STS, and STS with complex genetic alterations thereafter designed genetically-complex STS; p<2.2E-16, Fisher exact test). Cluster I (N = 97) consisted of all 29 SFTs in the left branch (cluster Ia) and 68 STSs in the right branch (cluster Ib). These 68 STS included all genetically-simple STSs (all 5 DFSPs, all 5 GISTs, all 20 myxoid/round cell liposarcomas, and all 6 synovial sarcomas), and only 32/143 genetically-complex STSs (19/46 dedifferentiated liposarcomas, 9/23 pleiomorphic liposarcomas, 3/35 leiomyosarcomas, and 1/39 malignant fibrous histiocytomas). Cluster II (N = 111) included only genetically-complex STSs, with leimyosarcomas (left branch, cluster IIa) being more homogeneous than dedifferentiated liposarcomas, pleiomorphic liposarcomas, and malignant fibrous histiocytomas (right branch, cluster IIb). The most heterogeneous STSss were the dedifferentiated liposarcomas and the pleiomorphic liposarcomas, which were scattered across the two clusters, intermixed with other STS subtypes. The pvclust package defined the robustness of tumor clusters (data not shown). As expected, all SFTs and all genetically-simple STSs, except GISTs (93% robust cluster), were included in 100% robust clusters. By contrast, only a part of genetically-complex STSs (from 26 to 66% according to the histological type) were included in robust clusters (AU p-value ≥95%).

Clustering identified coherent gene clusters (zoomed in Figure 1C as metagenes) corresponding to specific biological functions or cell types: a “proliferation cluster” overexpressed in genetically-complex STSs, a “muscle cluster” overexpressed in leimyosarcomas overall, an “immune cluster” and an “extracellular matrix cluster” overexpressed in dedifferentiated liposarcomas and malignant fibrous histiocytomas overall in agreement with their immune and fibrous features, a “ribosomal protein cluster” overexpressed in myxoid/round cells liposarcomas overall. Other biologically relevant clusters included a “vascular cluster” and an “early response gene cluster”. We also identified a cluster of co-expressed genes representing a presumptive gained chromosomal region – the “12q13–15 gain cluster” - of which 86% of genes, including *MDM2* and *CDK4*, are located on the 12q13–15 chromosomal region; as expected, its expression was visually associated with dedifferentiated liposarcomas. Visually, 4 gene clusters (proliferation, immune, muscle, and extracellular matrix) were underexpressed in SFTs overall as compared with genetically-complex STSs. Finally, we confirmed the expected overexpression of some genes: *KIT* and *ANO1/DOG1* in GISTs, *MDM2* and *CDK4* in non-myxoid/round cells liposarcomas, *CALD1* and tropomyosin genes (*TPM1*, *TPM2*) in leiomyosarcomas, *PDGFB* in DFSPs, *MUC1/EMA* in synovial sarcomas, and *CD34* in SFTs.

### Comparison of SFTs and All STSs by Supervised Analysis

**Figure 2:**
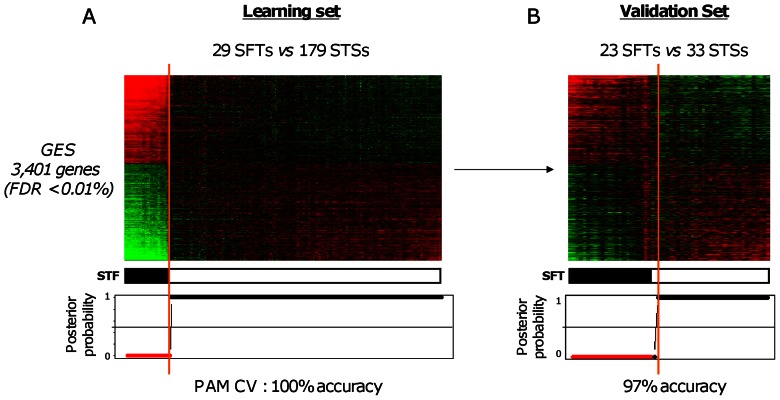
Supervised analysis of SFTs and all STSs. ***A.*** Classification of 208 samples (29 SFTs and 179 STSs) from the learning set using the 3,401-gene expression signature. *Top*, matrix of gene expression data. The legend is similar to Figure 1A. Tumor samples are ordered from left to right according to the decreasing correlation coefficient of their expression profile with the mean profile of the SFT samples. The genes are ordered form top to bottom according to their decreasing SAM statistics of SFT association. *Middle*: actual histological type (black, SFT; white STS). *Bottom,* probability (from 0 to 1) for each sample to be predicted as non-SFT by the PAM model based on the signature. Red dots represent SFT samples, and black STS samples. The solid orange vertical line indicates the threshold of 50% (equiprobability according to PAM model) that separates the two signature-predicted classes of samples. ***B.*** Similar to A, but applied to the 56 samples (23 SFTs and 33 STSs) from the independent validation set.

SAM analysis identified 3,401 genes as differentially expressed between SFTs (N = 29) and STSs (N = 179) from the learning set (FDR inferior to 0.01%), including 1,622 and 1,779 genes, respectively overexpressed and underexpressed in SFTs (Table S2). Figure 2A shows the resulting classification of 208 samples that, as expected, perfectly correlated with the pathological type (p<2.2E-16, Fisher’s exact test). The robustness of the Gene Expression Signature (GES) was first verified by PAM cross-validation in the learning set with 100% of samples correctly classified (p<2.2E-16, Fisher’s exact test). More importantly, the signature and the PAM model defined in the learning set, when applied to a totally independent validation set of 56 samples (23 SFTs and 33 STSs), nearly perfectly separated the two pathological types with only 1 STS sample falsely predicted as SFT (97% accuracy; p = 5.4E-16, Fisher’s exact test; Figure 2B).

To translate the RNA expression profiles into functionality, the list of differentially expressed genes was interrogated by IPA and GO software. Given the high percentage of differential genes, the gene list was first reduced to genes with an arbitrary fold change (FC) of expression between SFT and STS of at least 2 (702 genes). Results are shown in Table S3.

### Comparison of SFTs and Genetically-simple STS by Supervised Analysis

Because SFTs displayed whole-genome expression profiles closer to those of genetically-simple STSs than to those of genetically-complex STSs, themselves very different from genetically-simple STSs, we repeated the analysis by comparing in the learning set the 29 SFTs with the 36 genetically-simple STSs. The results were very close to those of the first analysis. A total of 2,914 genes were differentially expressed (1,368 and 1,546 respectively overexpressed and underexpressed in SFTs) with a FDR inferior to 0.01% (Table S4), with 2,052 genes common between the two signatures (60% of genes for the first signature, 70% for the second one). The signature was robust with 100% accuracy of classification (Figure S1) by the PAM model in the learning set by cross-validation (p = 4E-19, Fisher’s exact test) and 95% in the independent validation set (23 SFTs and 15 STSs) where only 2 out of 38 samples were misclassified (p = 8.8E-09, Fisher’s exact test).

Analysis of ontologies was applied to the 752 differential genes displaying a FC of expression between SFT and STS of at least 2 (Table S5).

### Transcriptional Alterations are not Due to DNA Copy Number Alterations

Twelve out of our 16 SFT samples had been previously profiled at the DNA level using whole-genome aCGH [32]. Regarding the 3,401-gene signature, 3.071 genes not located on sexual chromosomes were present on our aCGH chips. As shown in Table S6, only 11 out of 3,071 genes showed CNA in at least 1 out of 12 tumors: *ADAM22, AFTPH, HLA-DRB6*, *MEIS1*, *PELI1*, *RAB1A*, *SERTAD2*, *SLC25A40*, *SPRED2*, *SRI,* which were gained in only 1 tumor, and *GSTT1* in 2 tumors. However, the CNA was not necessarily the cause of RNA deregulation. Five of these 11 genes are located in chromosomal regions with known copy number variation (CNV). For three other genes, the tumor with gain did not show the highest expression level within the 12 samples. In fact, such positive correlation was observed for 3 genes only (*ADAM22*, *AFTPH, SRI*). Thus, most of the genes with RNA deregulation did not show any DNA CNA. Regarding the second signature (2,914 genes), the results were very similar (Table S7).

### Transcriptional Heterogeneity of SFTs and intra-SFT Supervised Analyses

As shown in Figure 1, SFTs constituted a homogeneous cluster when compared to STSs on a whole-genome scale. To study the intrinsic degree of transcriptional heterogeneity of SFTs, hierarchical clustering was applied to the 29 SFT samples from the learning set. Two major robust tumor clusters (AU p-value ≥95%) were identified (Figure S2); they did not correlate with the following histoclinical features: tumor location (meningeal *vs*. extra-meningeal), mitotic count (high *vs*. low), and histological subtype (cellular *vs*. conventional).

**Figure 3:**
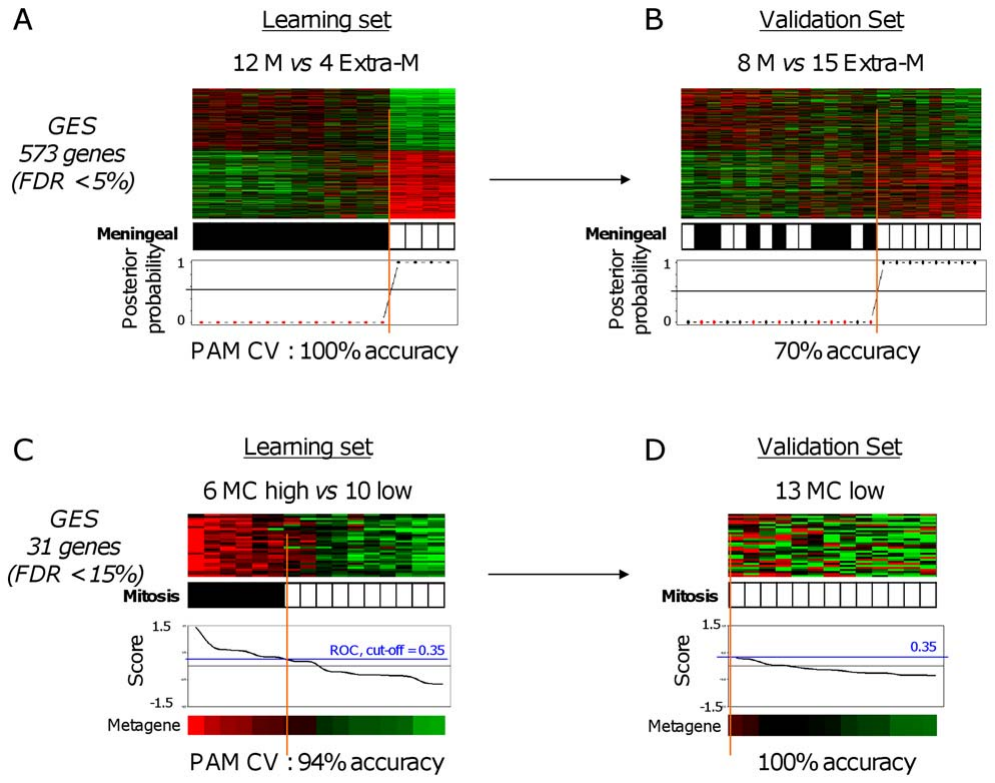
Supervised analyses of SFTs based on the location and mitotic count. ***A.*** Legend similar to Figure 2, but applied to 16 SFT samples (12 meningeal M and 4 extra-meningeal Extra-M). The signature includes 573 genes. Samples are ordered from left to right according to the decreasing correlation coefficient of their expression profile with the mean profile of the meningeal samples. The solid vertical line indicates the threshold of 50% (equiprobability according to PAM model) that separates the two signature-predicted classes of samples. The genes are ordered form top to bottom according to their decreasing SAM statistics of meningeal SFT association. ***B.*** Similar to A, but applied to the 23 samples from the independent validation set (8 meningeal M and 15 extra-meningeal Extra-M). ***C.*** Similar to A, but applied to 16 SFT samples (10 with low mitotic count MC and 6 with high count). The signature includes 31 genes. Samples are ordered from left to right according to the decreasing metagene score. The solid vertical line indicates the optimal cut-point (0.33) defined using ROC analysis that separates the two signature-predicted classes of samples. ***D.*** Similar to C, but applied to the 13 samples from the independent validation set (all with low mitotic count).

Supervised analyses were then done within our 16 SFTs (learning set), centered on two features, anatomic location and mitotic count. Regarding the location, we compared the profiles of 12 meningeal *versus* 4 extra-meningeal samples from our series. We did not include West’s samples because their anatomic location was not available. SAM identified 573 genes (FDR <5%) as differentially expressed (Table S8), that perfectly classified the 16 samples (Figure 3A). The robustness of this signature was confirmed by internal cross-validation, but above all, by external validation in the independent validation set (Hajdu’s set) with 70% accuracy in the resulting classification (p = 0.019, Fisher’s exact test; Figure 3B). IPA ontologies associated with the 573-gene list are shown in Table S9.

The second supervised analysis compared samples according to their mitotic count: 6 “high” cases *vs*. 10 “low” cases in the learning set (our series alone). We found 31 genes (FDR <15%) as differentially expressed (Table S10), all overexpressed in the “high” count cases (Figure 3C). The robustness of this 31-gene signature was confirmed in the West’s data set where all 13 samples (low mitotic count) were correctly predicted (Figure 3D). To translate the signature into functionality, the list of 31 overexpressed genes was interrogated by IPA software. As expected (Table S11), most of the overrepresented canonical pathways and biological functions were associated with cell cycle, cell proliferation, mitotic spindle organization, and DNA replication and repair.

### Validation of mRNA Deregulation Using IHC

**Figure 4:**
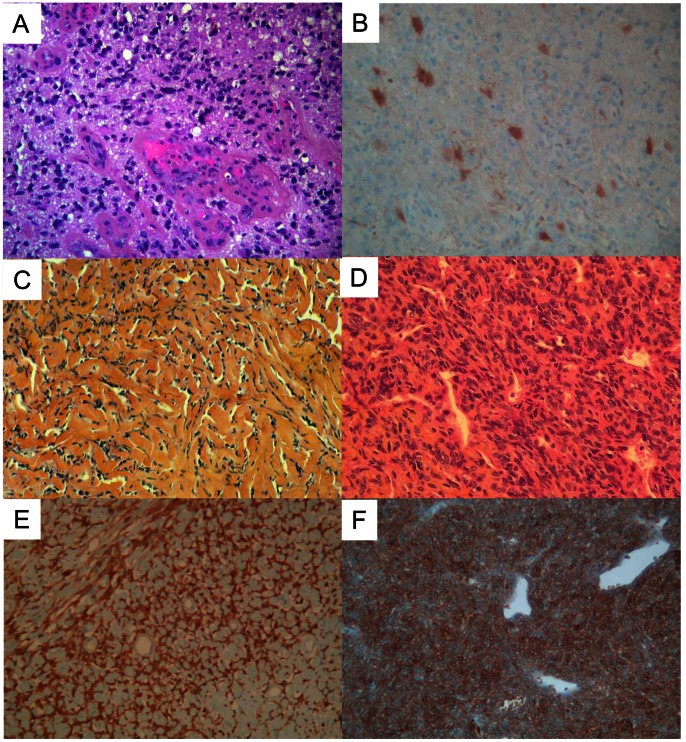
Microscopic aspects and ALDH1 expression using IHC. ***A.*** Microscopic features (HES) of a glioblastoma used as positive control for ALDH1 immunostaining. ***B.*** ALDH1 expression in the cytoplasm of few astrocytic tumor cells. ***C–D.*** Microscopic features (HES) of an SFT in a collagenic area (C) and of an SFT in a cellular area with an “hemangiopericytoma” vascular pattern (D). ***E–F.*** ALDH1 immunostaining in a collagenic area (E) and in a cellular area (F): note the strong and diffuse expression in the cytoplasm of tumor cells. For all images, magnification is ×25.

First, we sought to confirm at the protein level the differential expression of *ALDH1A1* between SFTs and STSs (FC superior to 30 at the RNA level). A total of 93 SFTs and 899 mesenchymal tumors, including 752 STSs, were analyzed on TMA. Examples of IHC staining are shown in Figure 4. We first verified the correlation between RNA (continuous values) and protein (semi-quantitative values representing the percent of stained tumor cells) expression in the 15 SFTs profiled with both techniques: 12 cases were IHC positive and 3 were negative. The correlation was significant (r = 0.60, p = 0.02, Pearson correlation). We then compared the protein expression in the whole series of SFTs and STSs. The difference was highly significant (p = 8.7E-83, Fisher exact test; OR = 334 [146–864]; Table 2) with 79 out of 93 SFTs that were positive (85%) *versus* only 12 out of 752 STSs (1.6%), thus confirming the transcriptional data. The number of stained tumor cells was relatively high within the 79 ALDH1-positive SFTs, with 14 cases scored as weak, but 26 as moderate (11 to 50% positive cells) and 39 as strong (>50% positive cells). All 44 dedifferentiated liposarcomas, all 339 pleomorph sarcomas, and all 71 leiomyosarcomas were negative. Seven out of 98 synovial sarcomas (7%) were positive, as were 4 out of 50 epithelioid sarcomas (8%). One out of 150 GISTs (0.6%) showed a weak positivity. Regarding the benign mesenchymal tumors, 19 out of 147 were positive (13%), including 18 out of 31 pleiomorph/fusiform cell lipomas (58%) and 1 out of 80 desmoId tumors (1.25%). The difference between the benign tumors and SFTs for ALDH1 expression was also significant (p = 1.1E-30, Fisher exact test; OR = 39 [18–92]);

**Table 2:** ALDH1 IHC status and histological types.

		ALDH1 IHC status		Total
		Negative	Positive	
**Histological type**	**STS**	740	12	752
	**STF**	14	79	93
**Total**		754	91	845

Second, we sought to confirm the differential expression of *AURKA* according to the mitotic index of SFTs. AURKA protein expression and mitotic index were simultaneously available for 51 out of 93 SFTs. Twenty-one out of 30 (70%) samples with low mitotic index were AURKA-negative and 14 out of 21 (67%) samples with high mitotic index were AURKA-positive (p = 0.01, Fisher exact test; OR = 4.51 [1.22–18.44]), thus confirming the transcriptional data.

## Discussion

To our knowledge, only one high-throughput molecular study in literature has compared the gene expression profiles of SFTs (N = 23) and STSs (N = 34) [22]; it revealed insights potentially relevant at the clinical level. Unfortunately, the series size impeded to test the robustness of differential expression profiles in an independent validation set, and no protein analysis was reported. In this context, our present analysis, which pools our own data set and 3 publicly available data sets, is the largest one analyzing the genome-wide transcriptional differences between SFTs and STSs and between relevant subgroups of SFT. We analyzed 52 SFTs and 212 STSs. This allowed the definition of independent learning and validation sets. Furthermore, the differential expression of some genes was confirmed at the protein level using IHC in a larger series of 93 SFTs and 752 STSs.

Whole-genome unsupervised analysis revealed that SFTs are very different from STSs and more homogeneous. SFTs were closer to genetically-simple STSs than to genetically-complex STSs. They displayed a transcriptional profile nearly as homogeneous as myxoid/round cell sarcomas, which are characterized by a specific translocation. The actual homogeneity of SFTs was better tested by clustering them separately on a whole-genome scale. Two robust clusters were identified, but did not correlate with any tested histoclinical features. To determine whether these robust clusters represent clinically relevant entities of SFT will require larger series.

The supervised analyses comparing SFTs and STSs identified a high proportion of differentially expressed genes despite a stringent FDR (<0.01%): 3,401 out of 10,089 tested genes (34%) in the comparison with all STSs, and 2,914 out of 10,089 (29%) in the comparison with genetically-simple STSs only. For comparison, the difference is as high as that we had reported between cancers of different anatomical origin (breast *vs* colon) or between acute leukemia representing different cell lineages (myeloid *vs* lymphoid) [24]. The reasons for this profound transcriptional difference between SFT and STS may include different cell–of-origin, but also particular SFT features such as lower proliferation rate overall, higher cell purity of samples (lesser contamination by non-tumor cells), absence of major genetic alterations and higher genome stability compared to STS. In a subset of samples previously profiled using aCGH [32], none of the differentially expressed genes showed recurrent DNA CNA in relation with mRNA deregulation, suggesting that CNA is not the responsible mechanism and that other causal molecular alterations, genetic and/or epigenetic, remain to be identified.

Importantly for high-throughput supervised analyses [37], the robustness of the two SFT/STS GES (SFTs *vs.* all STSs, and SFTs *vs.* genetically-simple STSs) was confirmed by the nearly perfect (97% and 95% respectively) classification of samples in independent validation sets. The two signatures were very similar: 70% of genes of the second GES were also present in the first GES. Our results also confirmed the previously reported overexpression of several genes in SFTs as compared with STSs, such as *BCL2* [10], and 10 out of the 16 discriminating genes quoted in the core text of Hajdu’s report [22]: *ALDH1A1*, *APOD*, *COL16A1*, *COL17A1*, *COL6A3*, *DDR1*, *ERBB2*, *FGFR1*, *GRIA2*, and *IGF1*. We also confirmed the strong overexpression of *IGF2*, responsible for hypoglycemia observed in the Doege-Potter syndrome and related to loss of imprinting (LOI) [22] by aberrant methylation. In this context, whether *IGF2* LOI is associated in SFT with global demethylation, as observed in Wilms tumors [38], warrants methylome analysis of SFT samples.

Given the fact that CD34, the most consistent marker of SFT to date, is a marker of hematopoïetic stem cells, it was quite intriguing to find *ALDH1A1*, another stem cell marker, among the genes most overexpressed in SFTs (fold change = 34). This overexpression was validated at the protein level in an independent and larger series of samples. Beside a potential diagnostic interest, such observation might have pathogenic significance. ALDH1 is a cytosolic detoxifying enzyme responsible for the oxidation of intracellular aldehydes [39] and involved in the retinoid metabolism with a role in early differentiation of stem cells, through the oxidation of retinol to retinoic acid [40]. It is recognized as a universal marker for both normal and cancer stem cells [41]. Mesenchymal cells are organized in a hierarchy in which ALDH1-positive cells display properties of stem cells [42]. Recently, the presence of increased ALDH1 activity allowed the identification of a subpopulation of human sarcoma cell lines [43]; [44] with stem cell properties, including high level of expression of stem cell genes such as *NANOG*, *OCT3/4*, *STAT3* and *SOX2*, and resistance to chemotherapy. Other results from our supervised analyses suggest some degree of stemness in SFTs: overexpression of retinoic acid receptors (*RARA*, *RARG*) and several *HOX* genes (*HOXA2-3-4*, *HOXB2-3-5-16*, *HOXC5*), known targets of retinoic acid, as well as overexpression of many genes previously identified as overexpressed in mesenchymal stem cells [45] such as *ANXA1*, *APOD*, *BMP2*, *CD44*, *CYR61*, *FOXO1*, *GLIPR1*, *CTGF*, *LIFR*, *SNAI2*, and *TGFB1* (data not shown). Ontology analysis (applied to genes differentially expressed and displaying a FC equal or superior to 2) confirmed this trend with the overrepresentation of ontologies related to stem cells within the genes upregulated in SFTs (“LXR/RXR activation”, “TR/RXR activation”, “LPS/IL-1 mediated inhibition of RXR function”, “PXR/RXR activation”, “ascorbate and aldarate metabolism”, “role of NANOG in mammalian embryonic stem cell pluripotency”, “NFkB signaling”, “WNT/beta-catenin signaling”, “HER2 signaling in breast cancer”, “growth hormone signaling”, “EGF signaling”).

The cell-of-origin of SFT is unknown. Initial studies suggested that pleural SFTs arise from immature mesenchymal stem cells located in the submesothelial layer of the visceral pleura [46]. More recent studies based on electron microscopy [47]; [48] showed considerable cellular heterogeneity in SFTs, which might originate from perivascular undifferentiated mesenchymal stem cells able to differentiate along several evolutional lines such as pericytic, endothelial and fibroblastic, following a pathway that occurs in normal angiogenesis. The frequent overexpression of ALDH1 and other progenitors and stem cells-related genes in SFTs agrees with this model and suggests an immature undifferentiated state with high number of mesenchymal progenitor/stem cells. Furthermore, since ALDH1 has been implicated in resistance to chemotherapy, notably to drugs classically used in sarcoma patients such as ifosfamide [49] and anthracyclines [44], its overexpression in SFTs could explain in part the chemoresistance of these tumors [50].

Interestingly, some genes overexpressed in SFTs encode therapeutic targets of drugs commercialized or under development (Table S2): *BCL2*, *CD33*, *EGFR*, *ERBB2*, *FGFR1, FNTA*, *FYN* and *YES1, RARA* and *RARG*, and *TLR3*. They also include several additional kinase genes such as *CHEK1*, *DDR1*, *EPHA1*, *EPHB1*, *JAK2*, *JAK3, LCK, PIK3C2G*, *PRKCD*, *PTK7*, *STK3*, and *STK4*, and 5 genes coding for histone deacetylases (*HDAC1*, *3*, *4*, *5* and *11*). Of course, before any clinical testing, functional experiments are warranted to determine whether the overexpression of these “druggable” genes in SFTs represents a “passenger” or a “driver” alteration. Histone deacetylases may be interesting candidates. HDACs are transcriptional corepressors, whose mutations and/or overepression have been reported in several cancers, making them new important therapeutic targets. Clinical trials with HDAC inhibitors are ongoing in sarcomas (NCT01112384: SB039; NCT00918489: vorinostat; NCT00878800: belinostat). On the basis of our observation, analysis of patients with SFT is awaited.

Our other supervised analyses compared expression profiles of SFT subgroups defined upon anatomical location and mitotic index. Meningeal SFTs have long been considered as different from pleural or extra-pleural SFTs. Our data suggest that they are not significantly different at the transcriptional level on a whole-genome scale. However, a robust 573-gene signature was identified by supervised analysis, suggesting differences between both locations. This result is consistent with that reported in a series of 23 samples [22]. Mitotic index is a prognostic feature of SFT, but displays some limitations at the technical (reproducibility) and prognostic levels. We identified a robust 31-gene signature discriminating SFTs with “low” *versus* “high” mitotic count. The analysis revealed many genes related to cell cycle and mitosis, including the classical Ki67 cell cycle marker and some kinases involved in G2 and M phases of the cell cycle: Aurora-A, a major kinase regulating mitosis, BUB1, BUB1B and TTK/MPS1 with known key roles in the various cell division checkpoints, and MELK, a regulator of the S/G2 and G2/M transitions. Interestingly, some of these genes such as *AURKA*, *BUB1*, *TTK*, and *RRM2* code for therapeutic targets of drugs under development (Table S5). Using IHC, we could validate the overexpression of *AURKA* in SFTs with high mitotic index in a 51-sample series.

In conclusion, we report the largest gene expression profiling study of SFTs. The robustness of our GES was confirmed using independent validation sets at the RNA level and for 2 genes at the protein level. The comparison between SFTs and STSs evidenced several differentially expressed genes, some of them could provide new diagnostic markers (*ALDH1*), as well as potential prognostic (*AURKA*) and/or therapeutic targets.

## Supporting Information

Figure S1 **Supervised analysis of SFTs and genetically-simple STSs.** ***A.*** Legend similar to Figure 2, but applied to 65 samples from the learning set including all 29 SFTs and the 36 genetically simple STSs. The signature includes 2,914 genes. ***B.*** Similar to A, but applied to the 38 samples from the independent validation set including all 23 SFTs and the 15 genetically simple STSs.
(PPT)

Figure S2 **Whole-genome expression profiles of SFTs.** ***A.*** Hierarchical clustering of 29 SFTs and 8,679 genes with significant variation in mRNA expression level across the samples (SD≥0.25). The legend is similar to Figure 1A. ***B.*** Dendrogram of samples. *Top*, two large groups of samples are evidenced by clustering and confirmed as robust by pvclust (AU p-value ≥95%). *Bottom*, some characteristics of samples are represented according to a color ladder: anatomic location (black, meningeal; white, extra-meningeal), histological type (black, cellular; white, conventional), and mitotic count (white, low; black, high).
(PPT)

Table S1 **Description of the gene expression data sets analyzed.**
(XLS)

Table S2 **List of 3,401 genes differentially expressed between SFTs** ***vs.*** **all STSs.**
(XLS)

Table S3 **Ontologies associated with the 3,401-gene list (SFTs** ***vs.*** **all STSs) restricted to 702 genes with FC ≥2.**
(XLS)

Table S4 **List of 2.914 genes differentially expressed between SFTs** ***vs.*** **genetically-simple STSs.**
(XLS)

Table S5 **Ontologies associated with the 2,914-gene list (SFTs** ***vs.*** **genetically-simple STSs) restricted to 752 genes with FC ≥2.**
(XLS)

Table S6 **Analysis of DNA CNA in the 3,071 genes differentially expressed between SFTs** ***vs*** **. all STSs, not located on sexual chromosomes and present on our Agilent aCGH chips.**
(XLS)

Table S7 **Analysis of DNA CNA in the 2,014 genes differentially expressed between SFTs** ***vs.*** **genetically-simple STSs, not located on sexual chromosomes and present on our Agilent aCGH chips.**
(XLS)

Table S8 **List of 573 genes differentially expressed between meningeal SFTs** ***vs.*** **extra-meningeal SFTs.**
(XLS)

Table S9 **Ontologies associated with the 573-gene list (meningeal SFTs** ***vs.*** **extra-meningeal SFTs).**
(XLS)

Table S10 **List of 31 genes differentially expressed between SFTs with high mitotic count** ***vs.*** **SFTs with low count.**
(XLS)

Table S11 **Ontologies associated with the 31-gene list (SFTs with high mitotic count** ***vs.*** **SFTs with low count).**
(XLS)
