# Supplementary material for: Gene Expression Profiling of Solitary Fibrous Tumors
Source: PLoS One. 2013 May 29;8(5):e64497. doi: 10.1371/journal.pone.0064497 (PMC3667191; doi:10.1371/journal.pone.0064497)
Supplement: Figure S1 — Supervised analysis of SFTs and genetically-simple STSs. A. Legend similar to Figure 2, but applied to 65 samples from the learning set including all 29 SFTs and the 36 genetically simple STSs. The signature includes 2,914 genes. B. Similar to A, but applied to the 38 samples from the independent validation set including all 23 SFTs and the 15 genetically simple STSs. (PPT) [file pone.0064497.s001.ppt]

## Slide 1
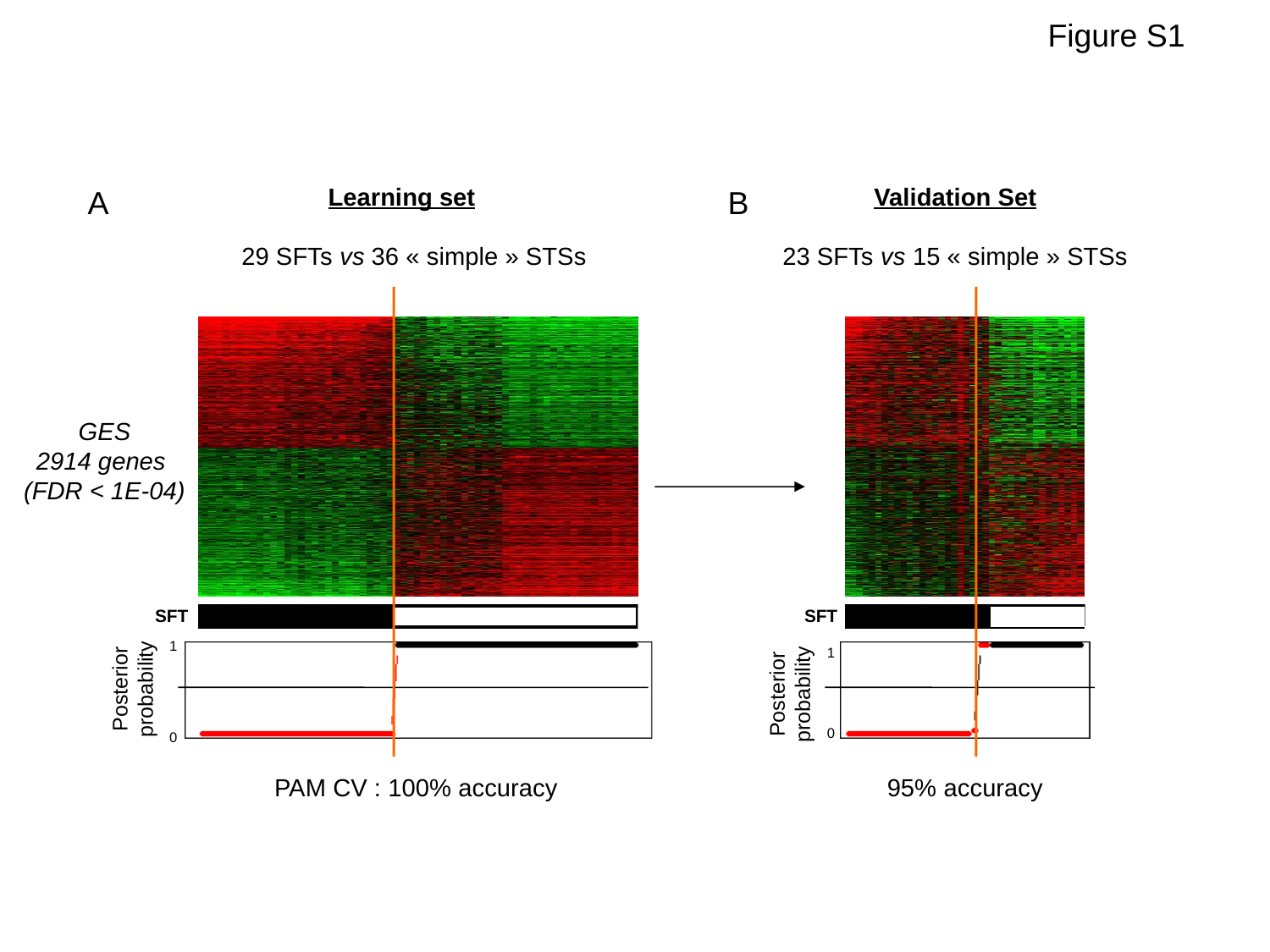

Figure S1
Learning set
29 SFTs vs 36 « simple » STSs
Validation Set
23 SFTs vs 15 « simple » STSs
A
B
GES
2914 genes
(FDR < 1E-04)
SFT
SFT
1
1
Posterior
probability
Posterior
probability
0
0
PAM CV : 100% accuracy
95% accuracy
