# Supplementary figures and images for: Gene Expression Profiling of Solitary Fibrous Tumors
Source: PLoS One. 2013 May 29;8(5):e64497. doi: 10.1371/journal.pone.0064497 (PMC3667191; doi:10.1371/journal.pone.0064497)

## Slide 1
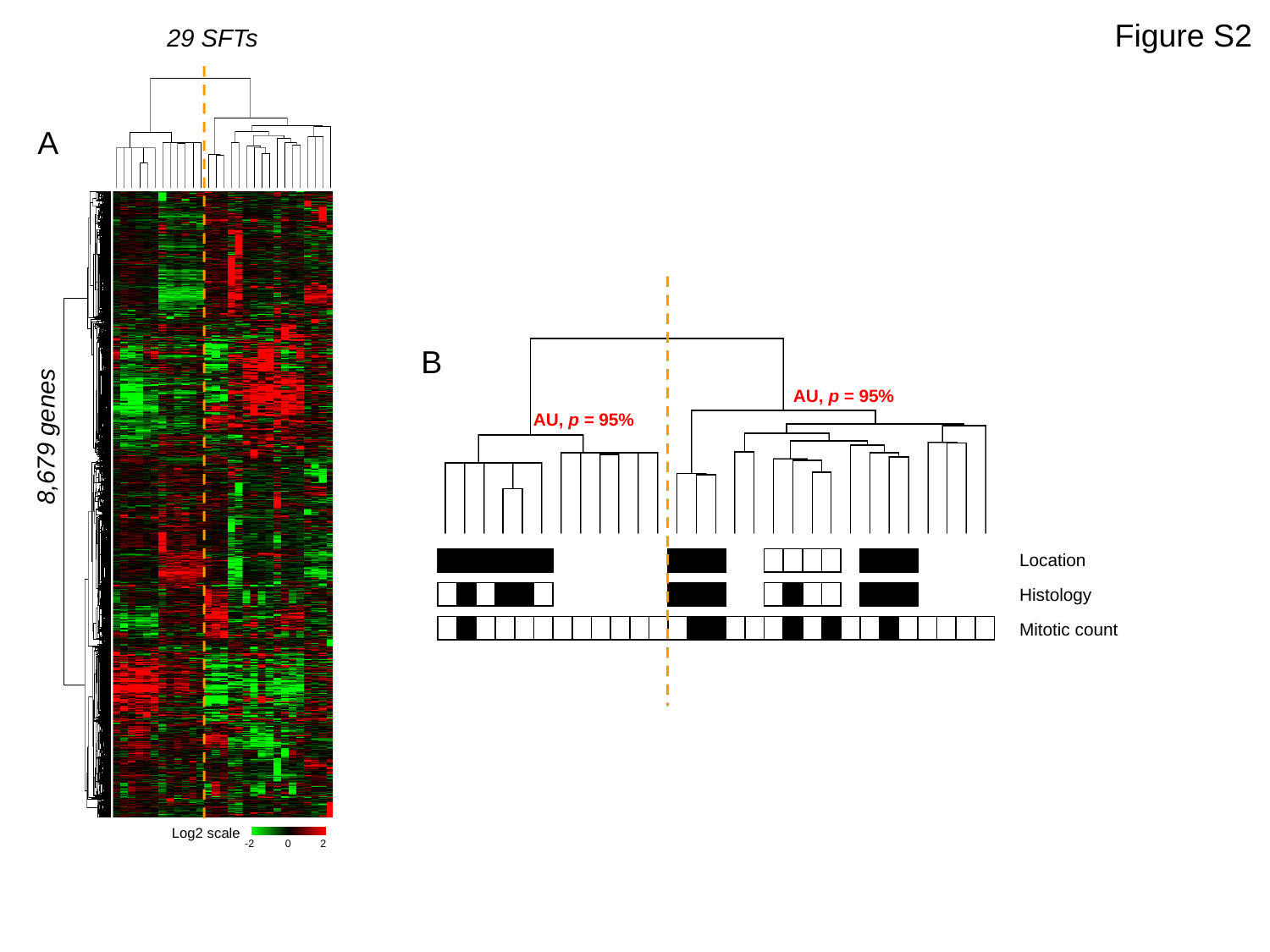

Figure S2
29 SFTs
A
Location
Histology
Mitotic count
B
 AU, p = 95%
 AU, p = 95%
8,679 genes
Log2 scale
-2
0
2

Supplement: Figure S2 — Whole-genome expression profiles of SFTs. A. Hierarchical clustering of 29 SFTs and 8,679 genes with significant variation in mRNA expression level across the samples (SD≥0.25). The legend is similar to Figure 1A. B. Dendrogram of samples. Top, two large groups of samples are evidenced by clustering and confirmed as robust by pvclust (AU p-value ≥95%). Bottom, some characteristics of samples are represented according to a color ladder: anatomic location (black, meningeal; white, extra-meningeal), histological type (black, cellular; white, conventional), and mitotic count (white, low; black, high). (PPT) [file pone.0064497.s002.ppt]
